# Tumor Volume as an Alternative Response Measurement for Imatinib Treated GIST Patients

**DOI:** 10.1371/journal.pone.0048372

**Published:** 2012-11-02

**Authors:** Gaia Schiavon, Alessandro Ruggiero, Patrick Schöffski, Bronno van der Holt, Dave J. Bekers, Karel Eechoute, Vincent Vandecaveye, Gabriel P. Krestin, Jaap Verweij, Stefan Sleijfer, Ron H. J. Mathijssen

**Affiliations:** 1 Department of Medical Oncology, Erasmus University Medical Center - Daniel den Hoed Cancer Center, Rotterdam, The Netherlands; 2 Department of Radiology, Erasmus University Medical Center, Rotterdam, The Netherlands; 3 Department of General Medical Oncology, University Hospitals Leuven, Catholic University Leuven, Leuven Cancer Institute, Leuven, Belgium; 4 Department of Trials and Statistics, Erasmus University Medical Center - Daniel den Hoed Cancer Center, Rotterdam, The Netherlands; 5 TNO Technical Sciences, The Hague, The Netherlands; 6 Department of Radiology, University Hospitals Leuven, Catholic University Leuven, Leuven, Belgium; 7 Breast Unit, The Royal Marsden Hospital, London, United Kingdom; Istituto di Ricerche Farmacologiche Mario Negri, Italy

## Abstract

**Background:**

Assessment of tumor size changes is crucial in clinical trials and patient care. We compared imatinib-induced volume changes of liver metastases (LM) from gastro-intestinal stromal tumors (GIST) to RECIST and Choi criteria and their association with overall survival (OS).

**Methods:**

LM from 84 GIST patients (training and validation set) were evaluated using manual and semi-automated Computed Tomography measurements at baseline, after 3, 6 and 12 months of imatinib. The ability of uni-dimensional (1D) and three-dimensional (3D) measurements to detect size changes (increase/decrease) ≥20% was evaluated. Volumetric response cut-offs were derived from minimally relevant changes (+20/−30%) by RECIST, considering lesions as spherical or ellipsoidal.

**Results:**

3D measurements detected size changes ≥20% more frequently than 1D at every time-point (*P*≤0.008). 3D and Choi criteria registered more responses than RECIST at 3 and 6 months for 3D-spheres (*P*≤0.03) and at all time-points for 3D-ellipsoids and Choi criteria (*P*<0.001). Progressive disease by 3D criteria seems to better correlate to OS at late time-points than other criteria.

**Conclusion:**

Volume criteria (especially ellipsoids) classify a higher number of patients as imatinib-responders than RECIST. Volume discriminates size changes better than diameter in GIST and constitutes a feasible and robust method to evaluate response and predict patient benefit.

## Introduction

Assessing anti-tumor effects of systemic therapy is particularly important to estimate activity of novel therapies in clinical trials, but it is also a key element to guide treatment changes in day-to-day routine cancer care. The Response Evaluation Criteria In Solid Tumors (RECIST), created in the late 1990s and updated in 2009, are currently considered as the ‘gold standard’ in most oncology settings [1]–[3]. They are based on uni-dimensional (1D) measurements (maximum diameter of target lesions) and their relative decrease or increase during therapy. Mathematically derived from the World Health Organization (WHO) criteria, which used the product of perpendicular cross-sectional diameters (bi-dimensional; 2D), RECIST is easier to use in daily clinical practice and is considered appropriate to assess tumor response in solid tumors [2], [3]. Therefore, RECIST has been adopted by academic institutes, cooperative groups, and industry for clinical trials with objective response or tumor progression as primary endpoints [1], [3].

However, with the recent improvements in technology both in radiology and informatics, a possible switch from anatomic 1D assessments of tumor burden to either functional assessment with Positron Emission Tomography (PET)/Magnetic Resonance Imaging (MRI) or volumetric (three-dimensional; 3D) assessments is a subject of debate [4]. At present, there is no validated alternative to RECIST, but the RECIST Working Group strongly encourages the development of novel markers/tools to predict potential therapeutic benefit for cancer patients [3].

**Figure 1:**
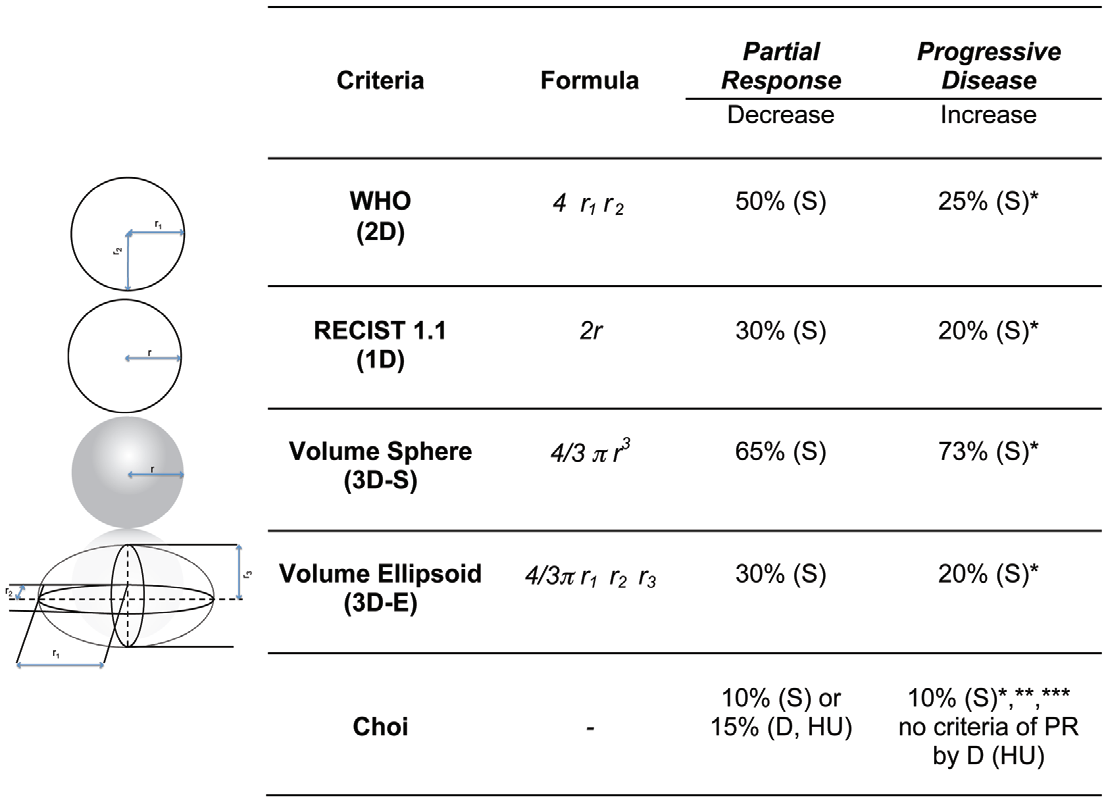
Size-based and Choi criteria. Different size-based criteria and Choi criteria with relative geometrically derived-cut-offs for partial response (PR) and progressive disease (PD). Corresponding geometrical shapes are shown at the left side of the figure. Stable disease (SD) corresponds to intermediate changes between PR and PD. *Abbreviations:* CT, computed tomography; D, tumor density; HU, Hounsfield unit; r, r_i_ (i = 1,2,3), radius; RECIST, Response Evaluation Criteria in Solid Tumors; S, size. *Symbols:* *appearance of a new lesion, **new intra-tumoral nodules or increase in the size of the existing intra-tumoral nodules, *** the sum of the longest diameters of target lesions as defined in RECIST [30].

A novel method to assess response to imatinib (Glivec®, Gleevec™) in gastrointestinal stromal tumors (GIST) patients was described by Choi *et al* [5]. ‘Choi criteria’ combines morphologic tumor response and biologic response (in terms of tumor density, as determined by measuring the Computed Tomography (CT) attenuation coefficient (Hounsfield units [HU]) [5], [6]. With the Choi criteria, smaller size changes than RECIST are sufficient to classify a size reduction as partial response (PR) (**Figure 1**). Choi criteria have been correlated to time to progression and disease specific-free survival [7]. However, other studies, comparing RECIST and Choi criteria in advanced GIST patients treated with sunitinib, found that discrimination of PR from stable disease (SD) with Choi criteria did not have prognostic value in terms of progression-free survival (PFS) and overall survival (OS) [8], [9]. Moreover, response evaluation according to RECIST sufficiently identified patients with a survival benefit merely by the absence of progressive disease (PD) [8].

The primary objective in this study was to develop a new approach for the assessment of volumetric changes of liver metastases in GIST during imatinib treatment. A well-defined, homogeneous single center cohort of patients (training set) was studied, and subsequently the same method was applied to an independent cohort from another institute (validation set).

We aimed to *1)* evaluate the ability of 3D and 1D measurements to discriminate size changes of liver lesions, *2*) compare volume, RECIST, and Choi criteria in early response evaluation, or lack of response, to treatment and *3)* determine the correlation of response according to these different criteria with OS.

As first study, we tested our hypothesis on one type of anatomical lesions (liver metastases) since they generally exhibit regular shapes and wide size ranges.

This is the first systematic approach with selection of two independent homogeneous populations to study the role of 3D measurements in response assessment in GIST.

## Patients and Methods

### Study Population

In order to test the hypothesis that volumetric measurements are more precise in assessing tumor response and detecting size changes than other criteria, we collected data and images from two independent populations: a training and validation cohort. The inclusion criteria for both cohorts were: histological diagnosis of GIST, liver metastases (with or without metastases in other sites), first-line imatinib therapy, and no previous (neo)adjuvant imatinib treatment. Imatinib was given until PD (assessed by RECIST) or unacceptable toxicity. The patients should have had standard contrast-enhanced CT scans at baseline, and at least one of the following time-points: 3, 6, and 12 months after the start of imatinib. In case of multiple available scans, the closest CT (in terms of number of days) to the corresponding time-point was chosen (**Table 1**). The study-design was retrospective, non-interventional and based on historically collected images and clinical data. The study was approved by the Institutional Review Board (commissie voor medische ethiek of University Hospitals of Leuven) at Leuven University Hospitals. The submission for this retrospective study to the Ethic Committee at Erasmus University Medical Center was not mandatory, since scans performed at the time of the diagnosis and during treatment were retrospectively retrieved, made anonymous and coded. No informed consent statement from patients was obtained, being retrospective. The Institutional Review Board in Leuven approved this procedure.

**Table 1:** Main clinical characteristics of patients in the training and validation cohorts.

	Training cohort (N = 56)	Validation cohort (N = 28)
Age at diagnosis, median (range) (ys)	61 (35–82)	57 (33–83)
Age at 1st imatinib, median (range) (ys)	64 (37–83)	58 (34–83)
Gender (M/F), n (%)	32/24 (57/43)	18/10 (64/36)
No subsequent therapy after 1st line imatinib, n (%)	31 (55)	15 (54)
2nd line therapy, n (%)	25 (45)	13 (46)
3rd lines of therapy and beyond (included Phase I), n (%)	4 (7)	7 (25)
Time between 1st imatinib – “3 mo CT”, median (range) (mo)	3.0 (2.3–3.6)	3.5 (2.8–3.7)
Time between 1st imatinib – “6 mo CT”, median, (range) (mo)	5.7 (5.2–6.4)	5.8 (5.5–6.7)
Time between 1st imatinib – “12 mo CT”, median (range) (mo)	11.8 (11.2–12.7)	12.2 (11.1–12.9)
Follow-up of patients still alive*, median (range) (mo)	42.3 (10.9–81.2)	72.5 (12.4–111.1)

Abbreviations: CT, Computed Tomography; F, female; M, male; mo, months; n, number; ys, years.

* calculated form the first day of therapy to the day the patient was last known to be alive.

OS was used as the primary outcome measure, as PFS was based on RECIST and consequently was not an independent variable. OS was calculated according to the landmark method, that is from the fixed time-point (date of CT) to the day of death (or last follow-up) [10]. For survival analyses, data collection was closed on July 20^th^ 2011 for both cohorts.

### Data Analysis

CT examinations were performed using multi-detector scanners (SOMATOM Sensation 64, 16, Plus 4 and Volume Zoom, Siemens AG; Philips Brilliance 64, Philips Medical System) with 3–5 mm section thickness and reconstruction intervals were studied. When patients were scanned with tri-phasic techniques, the portal-venous phase (70 seconds after administration of non-ionic iodinated contrast material) was studied.

Original Digital Imaging and Communications in Medicine (DICOM) files of all available CTs at the defined time-points were exported from a PACS workstation and images were anonymized. Manual measurements of all liver lesions were independently performed both by a medical oncologist (G.S.) and a radiologist (A.R.) by using the open source OsiriX® Imaging Software version 3.9 (Geneva, Switzerland) [11], [12]. Semi-automated measurements were acquired by the radiologist with the commercially available software ‘syngo CT Oncology®’ version 2009E (Siemens Medical Solutions, Inc.) using a Siemens Workstation [13]. In principle, this software is based on an extended version of the lung lesion segmentation approach previously described [14]. In that approach the density of lesions and surrounding parenchyma is known and can be used for the definition of a fixed threshold to separate them [14]. Such a discriminating technique may also be applied to liver lesions [15], [16]. The validation cohort was studied solely with semi-automated measurements, since they demonstrated robust correlation with manual measurements in our training cohort, which is consistent with literature data (**Figure S1**) [14]–[17].

### Manual and Semi-automated Measurements

After uploading the DICOM files in OsiriX® Imaging Software, manual measurements were performed according to our previously described method [18].

DICOM files were then uploaded in syngo CT Oncology® software. After displaying the segmentation results, lesions could be verified visually using the implemented 3D viewer, which provided multi-planar reconstructions (MPR) and orthogonal views [13]. Unsatisfactory segmentation results could be modified by assisting the segmentation process manually. Qualitatively insufficient segmentation after the third attempt was considered ´inadequaté and excluded from further analysis. The following parameters were documented: volume (mL), RECIST diameter (mm), mean HU (HU).

### Evaluation of Size Changes and Cut-offs for Response Assessment

According to Zhao *et al*, 1D and 3D tumor measurements were compared in two ways: ‘unadjusted/unscaled’ and ‘adjusted/scaled’ method [19].

With the unadjusted/unscaled method, the percentage changes of each measurement are directly compared [19]. Based on the literature, the ability of 3D and 1D measures to discriminate size changes (decrease or increase) ≥20% was compared [19], [20]. Comparisons of differences in measurements with the unadjusted/unscaled method identify clear disparities [19]. On the other hand, the adjusted/scaled method is a comparison of the measurements in assessing tumor response, thus after conversion of 1D measurements to an equivalent volume by using the standard published method [21], [22]. Considering geometrical formulas, a given percentage of change in diameter corresponds to a larger change in volume (Figure 1). These geometric correlations are determined by assuming that tumors are spherical and are the basis for the relationships between 1D (RECIST), 2D (WHO), and suggested volumetric response guidelines [2].

The ability of 1D and 3D measures to assess response was evaluated on up to two target lesions, defined according to RECIST 1.1 [1]. Classical 3D cut-offs (spheres) have been established and applied before and are based on the volume formulas of geometric objects, as was done from the transition from WHO criteria to RECIST criteria (Figure 1) [2], [22].

For example, if the RECIST diameter reduces by 30%, this implies a spherical volume reduction of 65%, indicating tumor response (Figure 1). If tumor lesions were always spherical, we would expect that 1D and 3D classical criteria perform in a similar manner in identifying response to treatment, given the direct relationship between RECIST and 3D-spheres’ cut-offs. In fact, the description of a tumor according to RECIST and to 3D-spheres should amount to the same description because the single degree of freedom of RECIST (the diameter) equals the single degree of freedom of a sphere (also its diameter).

However, there is evidence showing that solid bodies are better described by ellipsoidal than spherical shapes [23], supported by our observation that liver metastases in GIST patients can be either spherical or ellipsoidal and they can change morphology during imatinib therapy [24]. Since a model with only 1 degree of freedom is too constrained to capture such changes in morphology, we decided to explore the benefits of employing a volumetric description. The easiest alternative in the sense of mathematical description is offered by an ellipsoidal description, which has 3 degrees of freedom instead of 1. To establish our model, which we refer to as ‘3D-ellipsoids’, we had first to choose the type of ellipsoidal body and next to determine appropriate cut-off criteria.

Ellipsoids can be of three different types: 1) r_1_ = r_2_>r_3_ oblate spheroids (disk-shaped), 2) r_1_ = r_2_<r_3_ prolate spheroids (like a rugby ball), 3) r_1_≠r_2_≠r_3_ scalene ellipsoids (‘three unequal sides’), r_1_, r_2_, r_3_ being the radii of the three axes of the ellipsoid, whose formula for volume is 4/3 π r_1_ r_2_ r_3_. Prolate spheroids seem to be a suitable choice for our model, since they offer the possibility to maintain 2 axes fixed and to allow the largest axis to change size. Therewith we may capture the most significant volume changes and are able to relate the largest radius r_3_ to the RECIST radius.

To establish appropriate cut-off criteria we reasoned as follows. Assume that at a certain time point the RECIST diameter and the radius r3 (>r2 = r1) in the ellipsoidal description are equal and measured along the same axis. If the subsequent tumor changes occur only along this axis, the actual volume may change as little as 30% for a change in RECIST diameter of 30%. Requiring that our model provides the same result as RECIST in case tumor changes occur along one and the same axis, we choose the same cut-off criteria, or in other words, the cut-offs for ellipsoidal shapes correspond to 30% (PR) and 20% (PD). This reasoning explains the PR and PD values listed in Figure 1. Change (decrease or increase) in only one axis (r_3_) would be necessary and sufficient to create these values.

Responses by Choi criteria were defined according to the method (mean HU of the region of interest (ROI) semi-automatically obtained on the CT slice of the longest diameters and approved by the reader) [5]–[7]. The mean values of the absolute and relative changes in CT density of target lesions from the baseline to each time-point were computed [25].

### Statistical Analysis

Statistical analyses were performed with GraphPad Prism software version 5.0a (San Diego, CA) and Stata version 11.1 (College Station, Tx). Linear regression and Spearman rank correlation (*r*), whichever appropriate, were performed to assess the correlations between measurements of target lesions from two independent readers, and between volumes and diameters. Agreement between readers and techniques (manual and semi-automated) was evaluated with Bland-Altman plots [26]. Percentages of cases with size changes ≥20% assessed by 3D and 1D measures and response assessment (RECIST versus other criteria) were compared by an exact test of table symmetry (recommended for sparse tables). Comparisons with Choi criteria included patients with availability of semi-automated measurements (for HU). Associations with OS were determined by Log-rank test and illustrated by Kaplan-Meier survival curves [27]. Hazard ratios and 95% confidence intervals were estimated by Mantel-Haenszel method [28]. A two-sided *P*-value <0.05 indicated statistical significance.

## Results

### Patient Populations

The training cohort was selected from a total of 175 GIST patients treated at Erasmus University Medical Center from January 2001 until October 2010, partly participating in previously reported clinical trials [18], [29]–[32]. Among them, we identified 56 patients with liver metastases treated with first-line imatinib, availability of CT scans at baseline and at least one follow-up time-point.

The validation cohort was obtained from the Leuven Connective Tissue Oncology BioRepository (LECTOR), a single institution database for mesenchymal tumors, kept at the Leuven University Hospitals, Catholic University Leuven, Leuven Cancer Institute. Among 59 eligible patients with liver metastases from GIST, 28 patients had available CTs and were included in the analysis. Table 1 shows the main clinical characteristics of patients in both cohorts.

Semi-automated analysis was not technically feasible for 5 patients in the training set, leaving the following comparisons available: 50, 54, and 46 patients (manual method) and 45, 50, and 42 patients (semi-automated method) at 3, 6, and 12 months, respectively. Available comparisons (both methods) for the validation cohort were: 28, 27, and 25 patients at 3, 6, and 12 months, respectively.

### Robustness of Manual and Semi-automated Techniques

A strong correlation exists between the two readers for 1D and 3D manual measurements (R^2^ = 0.99, *P*<0.0001, n = 363 lesions) and a low mean inter-observer variability exists in the training cohort (0.2% for 1D and 3.2% for 3D) (data not shown). Correlation between diameters and volumes is high, both for manual and semi-automated measurements (*r = *0.97 and 0.98, *P*<0.0001; Figure S1A–B). The two methods provide clinically consistent similar measures with variability of 0.15% for 1D and 9.6% for 3D (n = 341 lesions, Figure S1C–D).

### Evaluation of Size Changes (Unadjusted/unscaled Method)

**Figure 2:**
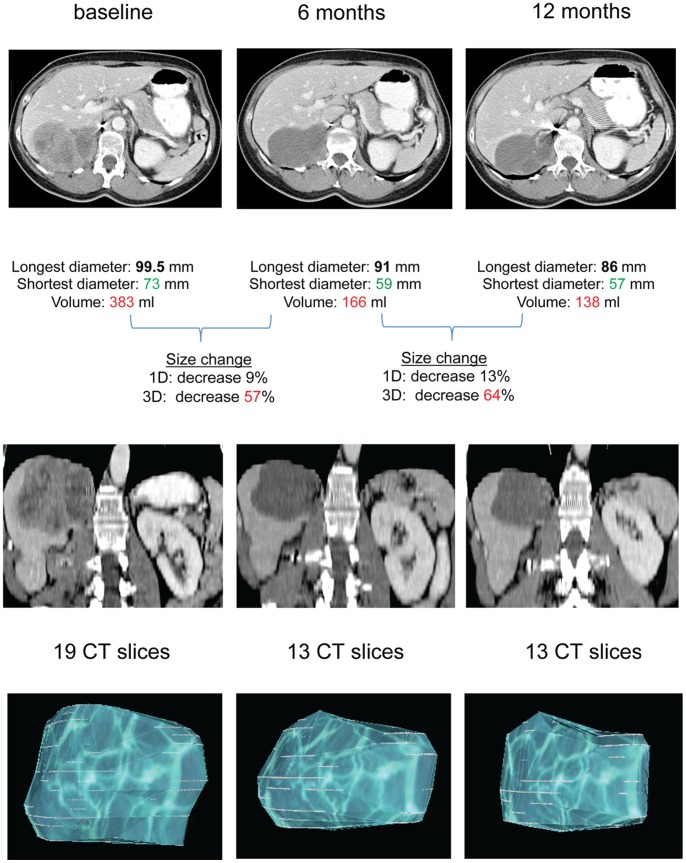
Example of manual and semi-automated images evaluation. This figure shows a case where 3D measurements could detect a size reduction >20%, and 1D measurements did not, because the shrinkage was mostly in the shortest diameters of the target lesion. To note, the shortest diameter (not taken into account by RECIST) decreased of 20% and 22% at 6 and 12 months respectively. Images are shown in the axial plane and in the coronal plan, and 3D reconstruction with OsiriX® Imaging Software is shown in the upper, middle, and lower part of the figure, respectively. The number of CT slices where the lesion could be identified at different time-points is indicated. Interestingly, this lesion was classified as SD according to RECIST and 3D-sphere criteria, and as PR according to 3D-ellipsoid and Choi criteria at both time-points.

A size change (decrease and increase) of ≥20% was detected more frequently by 3D measurements than 1D measurements at any-time point in the training cohort (P≤0.008, **Table 2**, **Figure 2**). The difference seemed to be higher at earlier time-points. In particular, 57% (16/28), 51% (18/35) and 18% (5/28) of the decreases detected with 3D measurements were classified by 1D measurements as “no change” at 3, 6 and 12 months, respectively. Among the discordant measurements, 3D and 1D detected changes in the same direction (decrease or increase of size) in 91% (50/55) of cases, where 96% of the cases showed that 3D measurements facilitate a superior identification of size changes (data not shown).

**Table 2:** Sensitivity in discrimination of size change # (%) (unadjusted/unscaled method).

			TRAINING COHORT					VALIDATION COHORT				
			Volume (3D)					Volume (3D)				
Time-Point (mo)		Change≥20%	Decrease	No	Increase	Tot	*P* *	Decrease	No	Increase	Tot	*P* *
**3**	**1D**	**Decrease**	12 (24)	1 (2)	1 (2)	14 (28)	***<.001***	7 (25)	–	–	7 (25)	***<.001***
		**No**	16 (32)	11 (22)	6 (12)	33 (66)		14 (50)	1 (4)	3 (11)	18 (64)	
		**Increase**	–	1 (2)	2 (4)	3 (6)		–	–	3 (11)	3 (11)	
		**Tot**	28 (56)	13 (26)	9 (18)	**50**		21 (75)	1 (4)	6 (21)	**28**	
**6**	**1D**	**Decrease**	17 (31)	1 (2)	–	18 (33)	***<.001***	12 (44)	1 (4)	–	13 (48)	**.** ***13***
		**No**	18 (33)	10 (19)	2 (4)	30 (56)		6 (22)	3 (11)	1 (4)	10 (37)	
		**Increase**	–	–	6 (11)	6 (11)		–	–	4 (15)	4 (15)	
		**Tot**	35 (65)	11 (20)	8 (15)	**54**		18 (67)	4 (15)	5 (19)	**27**	
**12**	**1D**	**Decrease**	23 (50)	–	–	23 (50)	**.** ***008***	15 (60)	–	–	15 (60)	**.** ***50***
		**No**	5 (11)	7 (15)	4 (9)	16 (35)		1 (4)	3 (12)	2 (8)	6 (24)	
		**Increase**	–	–	7 (15)	7 (15)		–	–	4 (16)	4 (16)	
		**Tot**	28 (61)	7 (15)	11 (24)	**46**		16 (64)	3 (12)	6 (24)	**25**	

Abbreviations: 1D, maximum diameter 1 D; mo, months; tot, total.

* 3D and 1D were compared in the ability to discriminate size changes (decrease and increase) ≥20% by an exact test of table symmetry, which is recommended for sparse tables.

In the validation cohort, a higher number of size changes ≥20% are detected by 3D measurements than by 1D measurements. This difference in terms of percentage reached significance after 3 months of therapy (P<0.001) (Table 2).

### Response Assessment (Adjusted/scaled Method)

To compare the criteria in assessing response, two different 3D cut-offs were used (Figure 1). A statistically significant difference in classification of response was found between RECIST and the other criteria at 3 and 6 months in the training cohort (*P*≤0.03, **Table 3**). In particular, an almost tripling of the PR was observed by using 3D-spheres as compared to RECIST and an even (much) higher number by using 3D-ellipsoids and Choi criteria. Also at 12 months these two criteria classified more PR than RECIST.

**Table 3:** Response assessment by different criteria # (%) (adjusted/scaled method).

TRAINING COHORT																	
			Volume spheres (S)					Volume ellipsoids (E)					Choi				
Time-point (mo)		RESPONSE	PR	SD	PD	Tot	*P* *	PR	SD	PD	Tot	*P* *	PR	SD	PD	Tot	*P* *
**3**	**RECIST**	**PR**	4 (8)	–	–	4 (8)		4 (8)	–	–	4 (8)		3 (7)	1 (2)	–	4 (9)	
		**SD**	6 (12)	34 (68)	1 (2)	41 (82)		22 (44)	15 (30)	4 (8)	41 (82)		23 (51)	11 (24)	4 (9)	38 (84)	
		**PD**	–	–	5 (10)	5 (10)		–	–	5 (10)	5 (10)		–	1 (2)	2 (4)	3 (7)	
		**Tot**	10 (20)	34 (68)	6 (12)	**50**	**.** ***03***	26 (52)	15 (30)	9 (18)	**50**	***<.001***	26 (58)	13 (29)	6 (13)	**45**	***<.001***
**6**	**RECIST**	**PR**	6 (11)	–	–	6 (11)		6 (11)	–	–	6 (11)		5 (10)	–	–	5 (10)	
		**SD**	10 (19)	30 (56)	–	40 (74)		24 (44)	13 (24)	3 (6)	40 (74)		23 (46)	14 (28)	1 (2)	38 (76)	
		**PD**	–	–	8 (15)	8 (15)		–	–	8 (15)	8 (15)		1 (2)	1 (2)	5 (10)	7 (14)	
		**Tot**	16 (30)	30 (56)	8 (15)	**54**	**.** ***002***	30 (56)	13 (24)	11 (20)	**54**	***<.001***	29 (58)	15 (30)	6 (12)	**50**	***<.001***
**12**	**RECIST**	**PR**	12 (26)	–	–	12 (26)		12 (26)	–	–	12 (26)		10 (24)	–	–	10 (24)	
		**SD**	3 (7)	17 (37)	3 (7)	23 (50)		13 (28)	5 (11)	5 (11)	23 (50)		13 (31)	7 (17)	2 (5)	22 (52)	
		**PD**	–	1 (2)	10 (22)	11 (24)		–	–	11 (24)	11 (24)		1 (2)	1 (2)	8 (19)	10 (24)	
		**Tot**	15 (33)	18 (39)	13 (28)	**46**	**.** ***25***	25 (54)	5 (11)	16 (35)	**46**	***<.001***	24 (57)	8 (19)	10 (24)	**42**	***<.001***
		**Response**	**PR**	**SD**	**PD**	**Tot**		**PR**	**SD**	**PD**	**Tot**		**PR**	**SD**	**PD**	**Tot**	
**3**	**RECIST**	**PR**	1 (4)	–	–	1 (4)		1 (4)	–	–	1 (4)		1 (4)	–	–	1 (4)	
		**SD**	–	24 (86)	–	24 (86)		16 (57)	5 (18)	3 (11)	24 (86)		21 (75)	2 (7)	1 (4)	24 (86)	
		**PD**	–	–	3 (11)	3 (11)		–	–	3 (11)	3 (11)		2 (7)	–	1 (4)	3 (11)	
		**Tot**	1 (4)	24 (86)	3 (11)	**28**	***1.00***	17 (61)	5 (18)	6 (21)	**28**	***<.001***	24 (86)	2 (7)	2 (7)	**28**	***<.001***
**6**	**RECIST**	**PR**	5 (19)	1 (4)	–	6 (22)		6 (22)	–	–	6 (22)		6 (22)	–	–	6 (22)	
		**SD**	3 (11)	14 (52)	–	17 (63)		11 (41)	5 (19)	1 (4)	17 (63)		15 (56)	2 (7)	–	17 (63)	
		**PD**	–	–	4 (15)	4 (15)		–	–	4 (15)	4 (15)		3 (11)	–	1 (4)	4 (15)	
		**Tot**	8 (30)	15 (56)	4 (15)	**27**	**.** ***63***	17 (63)	5 (19)	5 (19)	**27**	**.** ***001***	24 (89)	2 (7)	1 (4)	**27**	***<.001***
**12**	**RECIST**	**PR**	9 (36)	–	–	9 (36)		9 (36)	–	–	9 (36)		9 (36)	–	–	9 (36)	
		**SD**	1 (4)	9 (36)	1 (4)	11 (44)		5 (20)	4 (16)	2 (8)	11 (44)		9 (36)	1 (4)	1 (4)	11 (44)	
		**PD**	–	–	5 (20)	5 (20)		–	–	5 (20)	5 (20)		3 (12)	–	2 (8)	5 (20)	
		**Tot**	10 (40)	9 (36)	6 (24)	**25**	***1.00***	14 (56)	4 (16)	7 (28)	**25**	**.** ***03***	21 (84)	1 (4)	3 (12)	**25**	**.** ***001***

Abbreviations: mo, months; PR, partial response; SD, stable disease; PD, progression of disease; RECIST, Response Evaluation Criteria in Solid Tumors; tot, total.

* *P* values refer to the comparison with RECIST 1.1.

An exact test of table symmetry was used to compare the sensitivity in assessing response between RECIST and the other criteria. Comparisons related to Choi included only patients with availability of HU (semi-automated).

Regarding the validation cohort, only 3D-ellipsoids and Choi performed differently than RECIST (*P*≤0.03), classifying PR more frequently than RECIST at any time-point. Importantly, all criteria assessed a similar number of PD in both cohorts.

### Prediction of Survival

**Figure 3:**
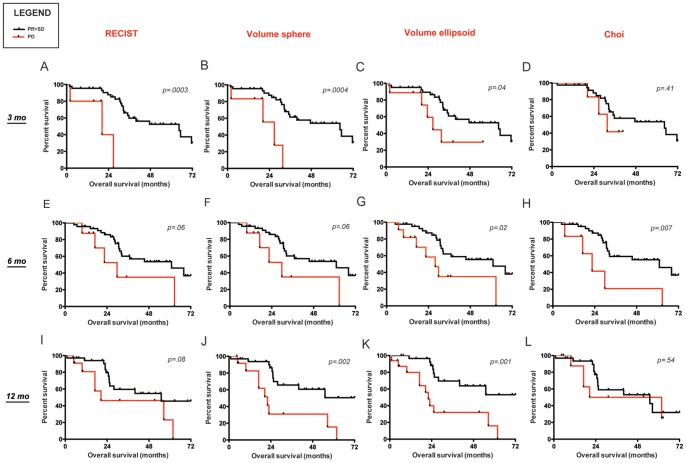
Overall survival by different criteria in the training cohort (2 curves). Overall survival (OS) shown up to 72 months (mo) of all assessable patients by response criteria, divided in progressed versus not progressed (SD+PR) at 3, 6 and 12 months of imatinib therapy. To note, survival curves related to Choi criteria include only patients assessable with both manual and semi-automated method, thus they should be evaluated separately and not directly compared with the other criteria (Panels D,H,L). **Panels A–C:** When the tumor response is evaluated on the basis of RECIST and 3D criteria, a statistically significant difference is observed in the long-term prognosis between patients with and without PD at 3 months. **Panels E–G:** Significant difference is observed in the long-term prognosis between patients with and without PD at 6 months only by 3D-ellipsoids. **Panels I–K:** A difference is observed in the long-term prognosis between patients with and without PD at 12 months only by 3D-spheres and 3D-ellipsoids, but no significant difference is observed by RECIST. **Panels D,H,L:** Significant difference is observed in the long-term prognosis between patients with and without PD by Choi criteria only at 6 months.

**Figure 4:**
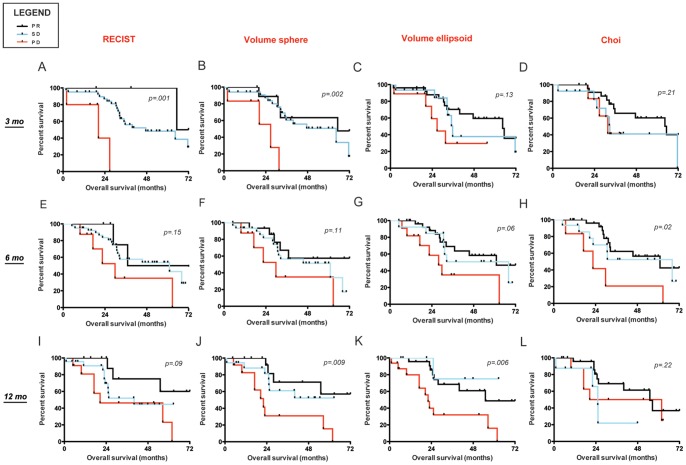
Overall survival by different criteria in the training cohort (3 curves). Overall survival (OS) shown up to 72 months (mo) of all assessable patients by response criteria, divided in PD versus SD versus PR, at 3, 6 and 12 months of imatinib therapy. To note, survival curves related to Choi criteria include only patients assessable with both manual and semi-automated method, thus they should be evaluated separately and not directly compared with the other criteria (Panels D,H,L).

**Figure 3** shows the ability of all the response criteria to predict survival in the training cohort at the studied time-points. When RECIST and 3D criteria were used in tumor response evaluations, significantly different OS was observed at 3 months between patients having a PD versus patients without a PD. At 6 months, only 3D-ellipsoids and Choi criteria appropriately predicted OS. Only 3D criteria (ellipsoids more than spheres) could significantly predict survival at 12 months. **Figure 4** shows the survival curves when patients are divided in three groups (SD, PR and PD).

Kaplan-Meier curves for the validation cohort are shown in **Figure S2 and S3**. Differences in OS were observed between patients with PD versus patients without PD at 6 and 12 months by using all the studied criteria.

## Discussion

In this study we demonstrated that volumetric criteria detect a size change of ≥20% in liver metastases more frequently than RECIST in imatinib-treated GIST patients, confirming previous findings on lung lesions [19]. When response criteria were applied, the results showed a significant difference, primarily in terms of response (not progression), showing that volume criteria (3D-spheres) classify a size reduction as PR more frequently than RECIST. 3D-ellipsoids were comparable to Choi criteria in assessing PR. These findings were anticipated, as the unadjusted scale does not apply geometrical corrections and thus reflects and maximizes absolute differences between measurement methods. However, the findings at the unadjusted scale show that even by taking into account the known geometric relationship between shapes (adjusted scale), different measurement methods do not necessarily produce equivalent results. Tumors do not always have a spherical shape or change in a spherical manner.

In addition, the feasibility of a simple semi-automated technique to assess tumor volume was shown.

Regarding survival, acknowledging the limitations of the relatively small sample size of the studied populations, it seems that volume measurements tended to be better associated with OS than RECIST.

Until now, there has been no alternative to anatomical 1D assessments of tumor burden validated on large sets of patients, although several limitations of 1D and 2D evaluations are widely known (e.g. irregular or confluent lesions, errors due to discrepant scan planes and patient positioning, intra−/inter-observer variability) [29], [33], [34] and an increasing awareness of the importance of their clinical implications is appearing [34]. As Gilles points out: “After all, for treatment outcomes, the RECIST classification is a convention, not an actual assessment of tumor burden. The key is the reliability of these metrics to minimize variability and ultimately reduce sample sizes” [35].

Volume changes were found to be more sensitive than 1D changes in phantom studies due to their wider dynamic range [36], [37]. Clinical studies on primary and secondary lesions concluded that, although more laborious and time-consuming, smaller changes in tumor burden may be detected earlier and more accurately by manual 3D measurements than by 1D and 2D measurements [33], [36], [37]. Moreover, semi-automated approaches make numerically large-scale volumetric quantification feasible, by offering time advantage, limited variability and improved reproducibility [38]–[40]. This is crucial for large multi-institutional trials where, for instance, the complex Choi criteria cannot be easily applied.

Interestingly, studies on computer-aided volumetric measurements identified circumstances and tumors (childhood cancers, mesothelioma, and GIST) where volumetric CT image analyses confer clear advantages in response assessment and additional prognostic information [19], [29], [33], [36], [37], [41], [42].

Due to the heterogeneity in the studied populations (e.g. different primary tumor, metastatic spread, treatment), studied lesions (e.g. lymph nodes, liver, lung metastases) and cut-off definitions, it is difficult to compare the data of previously published studies and draw general conclusions [19], [29], [41], [42]. Additionally, PFS has often been used in these studies as the endpoint for clinical outcome, which introduces the bias that PFS is estimated on the basis of RECIST and, consequently, PFS does not serve as an independent variable in this context.

Several years ago, Choi *et al.* succeeded in detecting responses earlier in GISTs, in line with PET but in contrast to RECIST readouts [5], [7]. However, Choi criteria are not yet incorporated into routine clinical practice or in Phase III oncology trials by regulatory agencies as primary method for disease assessment. This is probably due to their complexity, technical limitations (e.g. heterogeneity in hypoxic regions), and their unclear advantages to date [9], [43].

Nevertheless, novel criteria for response assessment in GIST (and solid tumors in general) may have important clinical consequences in drug development and routine patient care. An early and precise identification of response may limit the number of false-negatives in early-phase clinical studies, preventing the premature cessation of drug development. In routine clinical practice, early response assessment can have potential health economic benefits, given the high costs of innovative anticancer agents. Conversely, detecting an early lack of response may prevent unnecessary treatment continuation (and drug-related toxicity) in individual patients [44].

Although our volume criteria were tested only on liver metastases from GIST patients treated with a targeted agent, they could also be relevant for other sites of disease (e.g. peritoneal, primary) in GIST. Moreover, they will be tested in the response assessment to cytotoxic therapies in other tumor types, where the response depends more on morphological/dimensional changes than density changes. We will also prospectively incorporate the method based on our volume criteria as a response evaluation in addition to RECIST, in early clinical trials at our drug development unit.

Unfortunately, the lack of semi-automated data for some patients and the low number of patients in both cohorts prevent us from assessing the sensitivity of different methods in estimating PD, especially considering the long-term benefit typical for imatinib-treated GIST patients (median PFS of 21 months, irrespectively from mutation status) [30]. However, the population target was homogeneous and the relatively low incidence of the disease complicates patient accrual for larger study cohorts.

In summary, the initial results suggest that changes in tumor volume can be assessed as early as 3 months after initiation of imatinib treatment, whereas a lower magnitude of changes in diameters is seen during the same time period. This is due to the fact that the assumption that the tumor response will be comparable along all three tumor axes is not always correct [24].

Thus, 3D changes may have the potential to be a more sensitive and precise marker of regression or progression, although this should be demonstrated in prospective studies recruiting large populations. We are currently refining this method to discover the biologically optimal 3D cut-off to use in clinical studies for correlation with biomarkers and prognosis.

In fact, measurement change as a continuous variable is a technique increasingly considered as a way of better expressing a therapy’s antitumor activity.^33^

3D assessments could be a more rational strategy to define response criteria and corresponding cut-offs than the conventional criteria, which were based on an experiment of tumor measurements conducted by 16 clinicians, using solid spheres covered by foam rubber pads [45]. As Choi *et al.* suggested, now that we can measure the size of lesions to a precision of tenths of millimeters on cross-sectional images, the accuracy of current response criteria should be re-examined [5]. Therefore, this new method, incorporating volumetric evaluations of tumor response in metastatic GIST, has been shown to be very precise. In conclusion, although premature in terms of ability to set new cut-offs for response assessment criteria, our work is preliminary evidence that 3D measurements can be considered as a potential tool to observe response more efficiently than RECIST in solid tumors.

## Supporting Information

Figure S1 **Robustness of manual and semi-automated measurements.** Statistical analysis shows the robustness of the manual and semi-automated methods in the training cohort. Correlation between diameters and volumes for manual (**A**) and semi-automated (**B**) measurements (both P<0.0001; R^2^ = 0.97 and 0.98, respectively). Bland-Altman plots show the agreement between manual and semi-automated technique for diameters (**C**) and volumes (**D**) of target lesions. The two methods provide clinically consistent similar measures with variability of 0.15% and 9.6% for 1D and 3D respectively. *Abbreviation:* LoA, limits of agreement.
(TIF)

Figure S2 **Overall survival by different criteria in the validation cohort (2 curves).** Overall survival (OS) shown up to 72 months (mo) of all assessable patients by response criteria, divided in progressed versus not progressed (SD+PR) at 3, 6 and 12 months of imatinib therapy. **Panels A–D:** No significant difference is observed in the long-term prognosis between patients with PD and not PD at 3 months by any criteria. **Panels E–L:** Every criterion significantly divides patients with and without PD at 6 and 12 months.
(TIF)

Figure S3 **Overall survival by different criteria in the validation cohort (3 curves).** Overall survival (OS) shown up to 72 months (mo) of all assessable patients by response criteria, divided in PD versus SD versus PR, at 3, 6 and 12 months of imatinib therapy.
(TIF)
