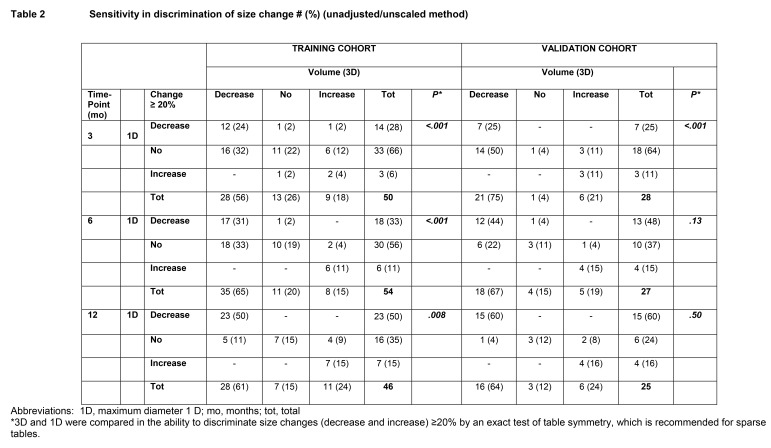# Correction: Tumor Volume as an Alternative Response Measurement for Imatinib Treated GIST Patients

**DOI:** 10.1371/annotation/0c66099f-d115-464e-95f3-f3b9412a325a

**Published:** 2013-01-17

**Authors:** Gaia Schiavon, Alessandro Ruggiero, Patrick Schöffski, Bronno van der Holt, Dave J. Bekers, Karel Eechoute, Vincent Vandecaveye, Gabriel P. Krestin, Jaap Verweij, Stefan Sleijfer, Ron H. J. Mathijssen

There are errors in Table 2. The correct table can be viewed here: 

**Figure:**