# Supplementary figures and images for: Tumor Volume as an Alternative Response Measurement for Imatinib Treated GIST Patients
Source: PLoS One. 2012 Nov 2;7(11):e48372. doi: 10.1371/journal.pone.0048372 (PMC3487791; doi:10.1371/journal.pone.0048372)

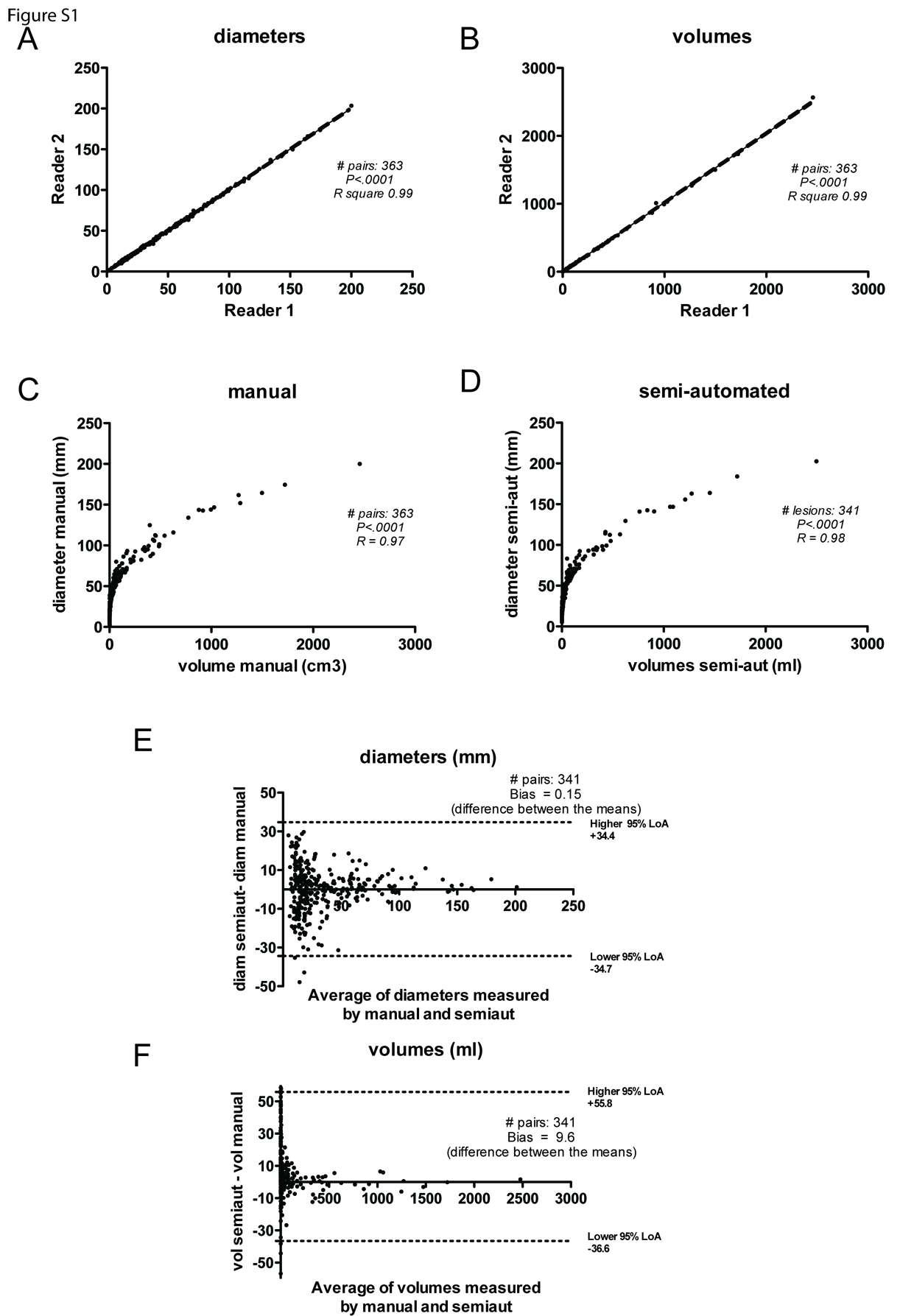

Supplement: Figure S1 — Robustness of manual and semi-automated measurements. Statistical analysis shows the robustness of the manual and semi-automated methods in the training cohort. Correlation between diameters and volumes for manual (A) and semi-automated (B) measurements (both P<0.0001; R2 = 0.97 and 0.98, respectively). Bland-Altman plots show the agreement between manual and semi-automated technique for diameters (C) and volumes (D) of target lesions. The two methods provide clinically consistent similar measures with variability of 0.15% and 9.6% for 1D and 3D respectively. Abbreviation: LoA, limits of agreement. (TIF) [file pone.0048372.s001.tif]

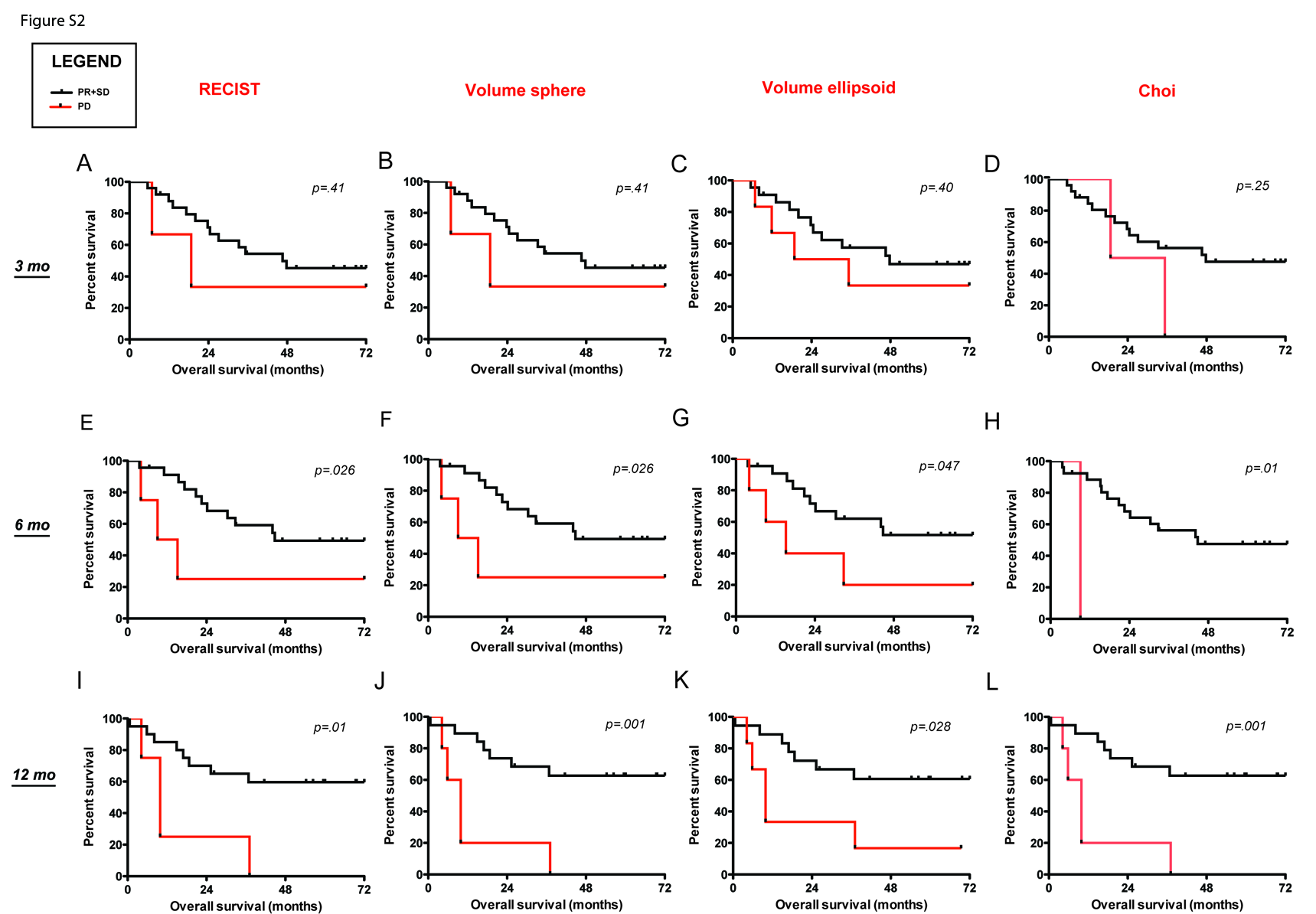

Supplement: Figure S2 — Overall survival by different criteria in the validation cohort (2 curves). Overall survival (OS) shown up to 72 months (mo) of all assessable patients by response criteria, divided in progressed versus not progressed (SD+PR) at 3, 6 and 12 months of imatinib therapy. Panels A–D: No significant difference is observed in the long-term prognosis between patients with PD and not PD at 3 months by any criteria. Panels E–L: Every criterion significantly divides patients with and without PD at 6 and 12 months. (TIF) [file pone.0048372.s002.tif]

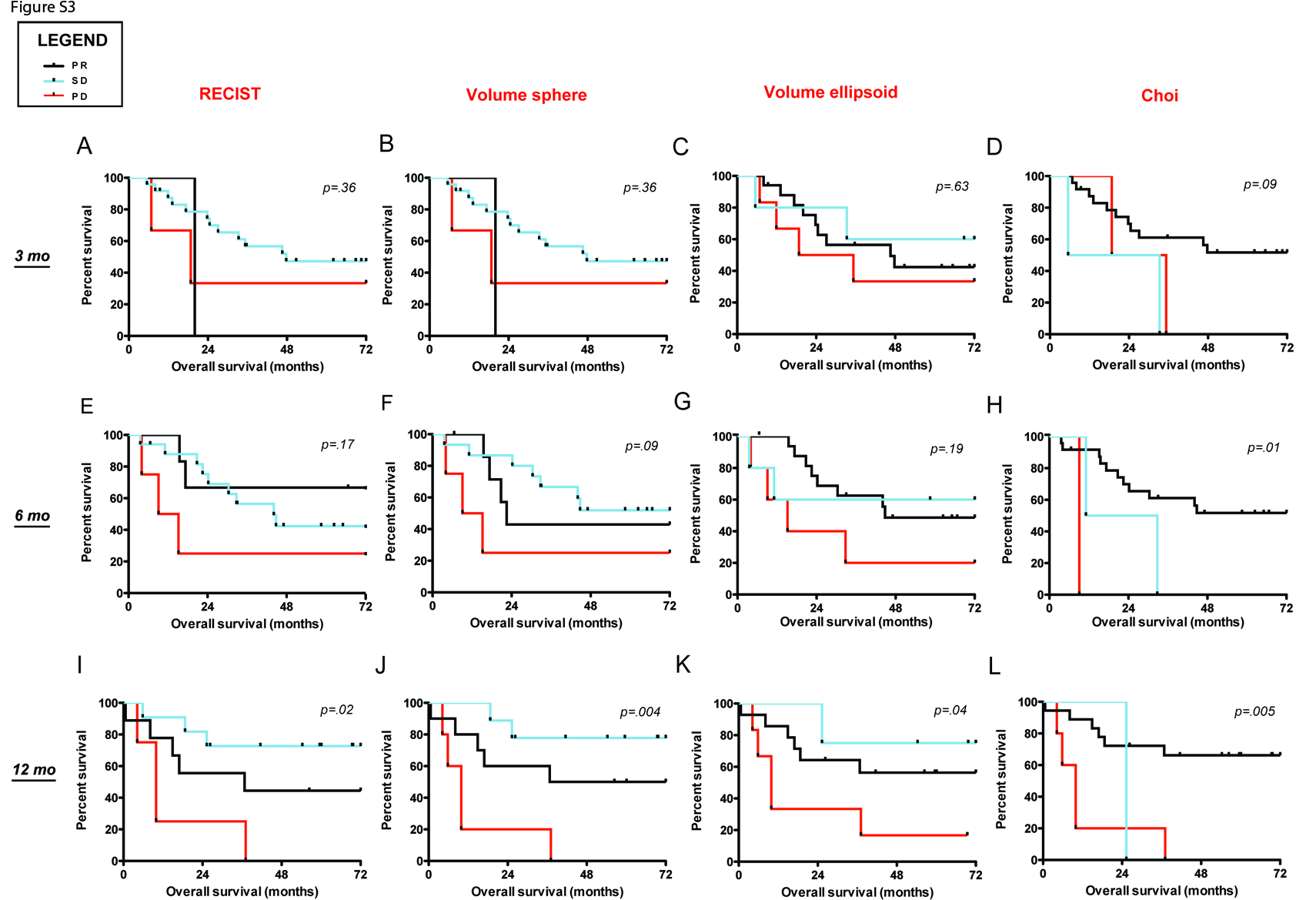

Supplement: Figure S3 — Overall survival by different criteria in the validation cohort (3 curves). Overall survival (OS) shown up to 72 months (mo) of all assessable patients by response criteria, divided in PD versus SD versus PR, at 3, 6 and 12 months of imatinib therapy. (TIF) [file pone.0048372.s003.tif]
